# Peroral pancreatoscopy without a guidewire for intraductal papillary mucinous neoplasm

**DOI:** 10.1055/a-2408-8685

**Published:** 2024-09-19

**Authors:** Haruo Miwa, Kazuki Endo, Shotaro Tsunoda, Ritsuko Oishi, Yuichi Suzuki, Hiromi Tsuchiya, Shin Maeda

**Affiliations:** 1 26437 Gastroenterological Center, Yokohama City University Medical Center, Yokohama, Japan; 2 Department of Gastroenterology, Yokohama City University Graduate School of Medicine, Yokohama, Japan

Peroral pancreatoscopy (POPS) for intraductal papillary mucinous neoplasm (IPMN) facilitates the detection of the mural nodules 1 2 . However, guidewire seeking prior to POPS causes erythema inside the pancreatic duct, which may lead to misdiagnosis. A novel slim cholangioscope (9-Fr eyeMAX; Micro-Tech, Nanjin, China) has the advantages of easy insertion and high mobility in the bending section 3 4 . Here, we report a case of POPS without a guidewire for IPMN ( Video 1 ).

Peroral pancreatoscopy without a guidewire in a case of intraductal papillary mucinous neoplasm facilitated an accurate preoperative diagnosis.Video 1

A 76-year-old-man was referred to our hospital because of dilation of the main pancreatic duct. As magnetic resonance cholangiopancreatography or endoscopic ultrasound could not detect mural nodules ( Fig. 1 ), we planned to perform POPS without a guidewire.

**Fig. 1:**
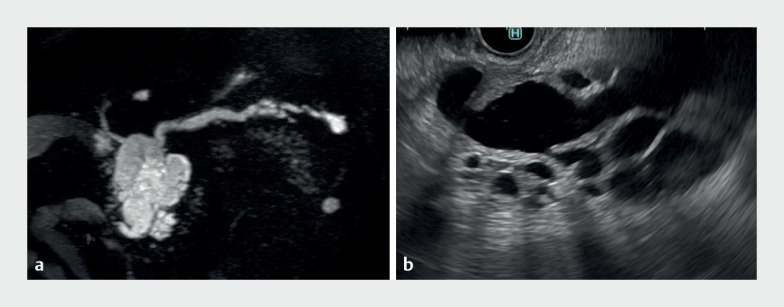
A 76-year-old man was referred with intraductal papillary mucinous neoplasm. **a** Magnetic resonance cholangiopancreatography revealed a pancreatic cyst with main pancreatic duct dilation. **b** Endoscopic ultrasound showed no mural nodules in the main pancreatic duct.

The orifice of the pancreatic duct was sufficiently dilated, and mucus flowed out. First, the catheter was inserted just over the papilla and the contrast agent was injected up to the pancreatic neck. Subsequently, a 9-Fr eyeMAX was inserted without a guidewire into the dilated main pancreatic duct ( Fig. 2 **a** ). Papillary mural nodules were observed in the main pancreatic duct of the pancreatic head ( Fig. 3 **a, b** ). Given the acute angle of the pancreatic duct between the pancreatic head and body, the eyeMAX was carefully inserted by the assistant with an angle maneuver. The tip of the eyeMAX was easily advanced to the pancreatic tail end without a guidewire ( Fig. 2 **b** ). No focal erythema or mural nodules were observed within the pancreatic duct in the body and tail ( Fig. 3 **c, d** ).

**Fig. 2:**
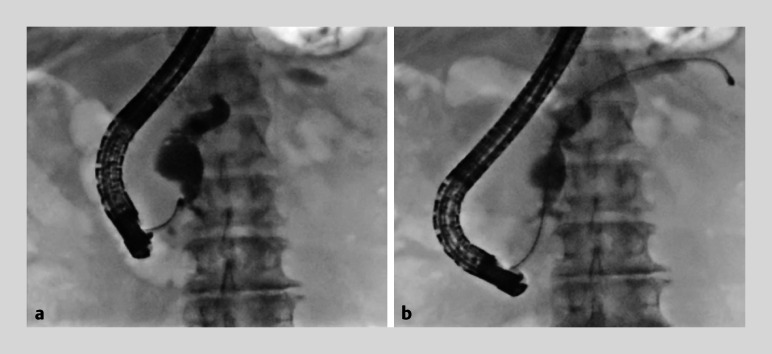
Fluoroscopic images of peroral pancreatoscopy with 9-Fr eyeMAX (Micro-Tech, Nanjin, China). **a** The main pancreatic duct in the pancreatic head was dilated, and eyeMAX was inserted without a guidewire. **b** EyeMax was advanced to the tail end.

**Fig. 3:**
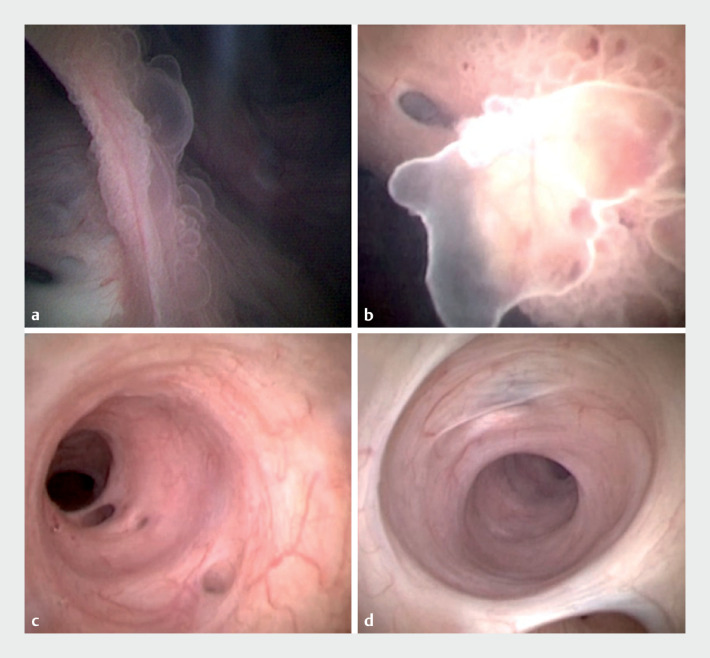
Images of peroral pancreatoscopy. **a, b** Mural nodules in the pancreatic head. **c** Focal erythema was not observed in the pancreatic body. **d** EyeMax reached the pancreatic tail end.

The pancreatic duct in the head was filled with mucus, and various forms of mural nodules were observed. EyeMAX was easily inserted into the branched duct, and mural nodules were detected. Negative biopsies were performed in several areas of the body and tail. Finally, targeted biopsies were performed in the pancreatic head.

The patient was discharged 2 days later without any complications.

Endoscopy_UCTN_Code_TTT_1AR_2AD

## Notes

Endoscopy E-Videos https://eref.thieme.de/e-videos
*E-Videos* is an open access online section of the journal *Endoscopy* , reporting on interesting cases and new techniques in gastroenterological endoscopy. All papers include a high-quality video and are published with a Creative Commons CC-BY license. Endoscopy E-Videos qualify for HINARI discounts and waivers and eligibility is automatically checked during the submission process. We grant 100% waivers to articles whose corresponding authors are based in Group A countries and 50% waivers to those who are based in Group B countries as classified by Research4Life (see: https://www.research4life.org/access/eligibility/ ). This section has its own submission website at https://mc.manuscriptcentral.com/e-videos .
